# Response to chemotherapy combined with anlotinib plus anlotinib maintenance in intra-abdominal desmoplastic small round cell tumors (IADSRCT): a case report and literature review

**DOI:** 10.1186/s12876-022-02463-y

**Published:** 2022-08-17

**Authors:** Ke Cheng, Xia Liu, Ye Chen, Kexun Zhou, ZhiPing Li

**Affiliations:** Department of Abdominal Oncology, Cancer Center of West China Hospital, Sichuan University, No. 37, GuoXue Xiang, Chengdu, 610041 Sichuan China

**Keywords:** Intra-abdominal desmoplastic small round cell tumor, Chemotherapy, Anlotinib

## Abstract

**Background:**

Intra-abdominal desmoplastic small round cell tumors (IADSRCT) are rare and aggressive neoplasia that are resistant to chemotherapy. Anlotinib is an oral multi-target tyrosine kinase inhibitor that also has anti-angiogenic and anti-proliferative properties. In this article, we report on a case showing effective and durable responses to chemotherapy combined with anlotinib in a young man with IADSRCT.

**Case presentation:**

A 27-year-old man was admitted to our hospital complaining of a palpable periumbilical mass that had been present for longer than 4 months. The diagnosis of IADSRCT was confirmed by biopsy and immunohistochemistry. An extensive unresectable metastasis was found on the initial diagnosis. The patient received six cycles of chemotherapy combined with anlotinib, and maintenance therapy with anlotinib was recommended. Hematochezia, proteinuria and hypertension were observed, however, long-term maintenance therapy was well tolerated. A partial response was observed after two cycles of combined therapy and the patient was still alive with stable disease at the time of reporting.

**Conclusions:**

Chemotherapy combined with anlotinib plus anlotinib maintenance showed promising efficacy and manageable toxicity in the treatment of advanced IADSRCT.

## Background

In 1991, Gerald et al., reported a case series in 19 patients with intra-abdominal desmoplastic small round cell tumor (IADSRCT) that were characterized as rare soft-tissue sarcomas (STS) with intra-abdominal locations. The tumors showed nesting patterns of growth with focal rhabdoid features and highly aggressive behaviors [1]. Multidisciplinary treatment is the main approach in the management of desmoplastic small round cell tumors (DSRCT) that includes chemotherapy, surgical resection, and radiation therapy. For patients with advanced/metastatic DSRCT, the efficacy of combined chemotherapy has been demonstrated in several clinical studies. Kushner et al. showed that a chemotherapy regimen (P6 protocol) including vincristine, doxorubicin, cyclophosphamide, ifosfamide and etoposide could achieve a response rate of 100% in patients with DSRCT [2]. Bertuzzi demonstrated that induction chemotherapy (ifosfamide, epirubicin, and vincristine) followed by high-dose chemotherapy produced an overall response of 43% in DSRCT [3]. However, high levels of toxicity continue to limit the use of combined therapy. The development of multidisciplinary treatments has resulted in five-year overall survival rates to DSRCT of 15–30% [4]. There is an urgent need to explore novel therapies that are effective and well-tolerated towards improving patient outcomes in DSRCT.

Anlotinib is an oral, multi-target, tyrosine kinase inhibitor (TKI) that targets VEGFR-2 and -3, FGFR1-4, PDGFR-α and -β, c-Kit and Ret [5]. Based on the results of a phase II trial (NCT01878448), anlotinib is a landmark success in the treatment of refractory metastatic STS and was approved by the Chinese National Medical Products Administration for patients with STS with progressive disease after one line of anthracycline chemotherapy [6]. Also, the survival benefits of anlotinib combined with chemotherapy have been observed in patients with advanced STS and it may also have an important role in maintenance therapy. Wang et al. retrospectively analyzed patients with advanced/metastatic STS who received chemotherapy combined with anlotinib plus anlotinib maintenance therapy. This regimen achieved an objective response rate (ORR) of 34% and a disease control rate (DCR) of 69% [7]. Another study by Liu et al. revealed that switch maintenance therapy with anlotinib provided a remarkable DCR of 81.0% for patients with unresectable or metastatic STS who had previously responded to chemotherapy [8].

Based on these findings, the novel combination provides an alternative treatment for this patient population. However, the combination of chemotherapy and anlotinib in IADSRCT remains unclear. In this article, we report a case study of IADSRCT that was treated with combination therapy and include a systematic review of the literature concerning this rare disease.

## Case presentation

**Fig. 1:**
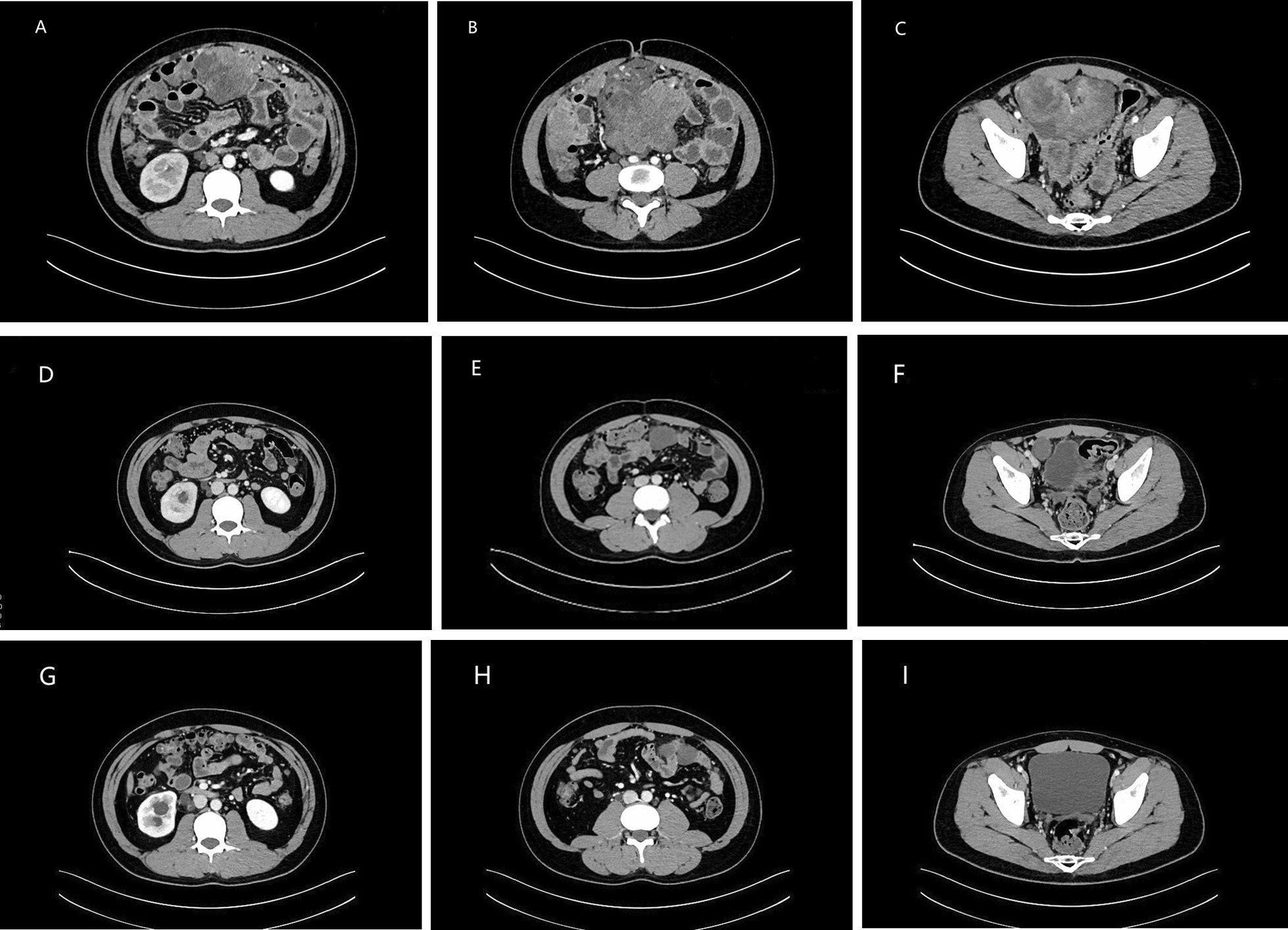
Abdominal CT showing the radiological response to combined and maintenance therapy. **A**–**C** Multiple masses in the pelvic and abdominal cavities at the initial diagnosis. **D**–**F** Partial response was achieved after two cycles of combined therapy. **G**–**I** Continuous partial response during maintenance therapy with anlotinib

**Fig. 2:**
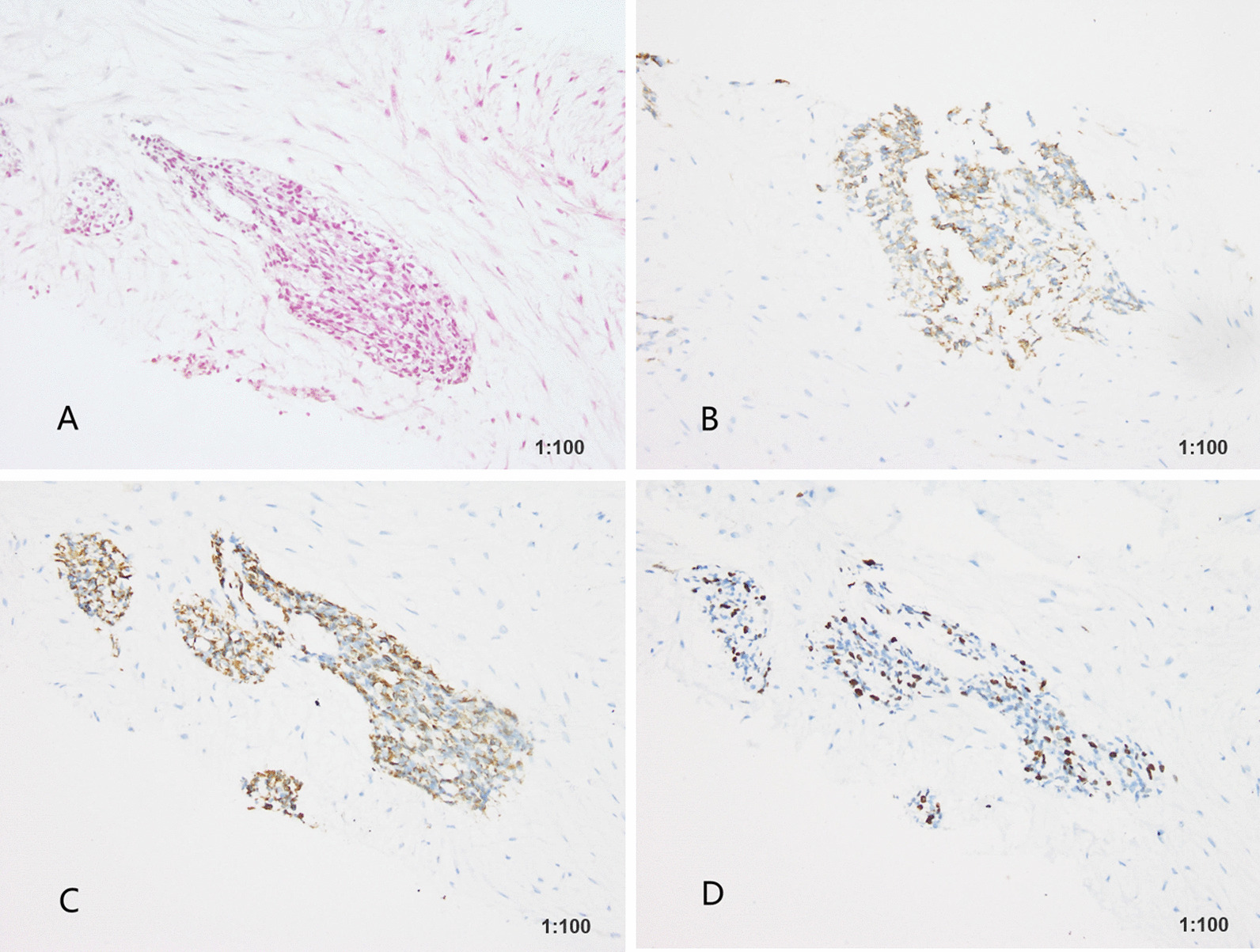
Histological and immunohistochemistry analysis of the intra-abdominal desmoplastic small round cell tumor. **A** Histological appearance (× 200 magnification). **B** Immunohistochemistry of CD56 (× 200 magnification). **C** Immunohistochemistry of DES (× 200 magnification). **D** Immunohistochemistry of Ki-67 (× 200 magnification)

In May 2019, a 27-year-old man visited our hospital complaining of a palpable periumbilical mass that had been presented for longer than 4 months. Enhanced computer tomography (CT) of the abdomen showed multiple masses in the pelvic and abdominal cavities that involved the intestine, liver, and ureter. Multiple lymph node metastases were also identified with moderate ascites (Fig. 1A–C). CT-guided biopsy suggested by multiple disciplinary team was performed and revealed DSRCT and immunohistochemistry test showed Des (+), CD56 (+), EMA (−), CK7 (−), SMA (−), S-100 (−), Ki-67/MIB-1(+, 20%), supporting the diagnosis of IADSRCT (Fig. 2). As complete resection could not be obtained due to the extensive metastasis, systemic therapy was recommended by a multidisciplinary team (MDT).

**Fig. 3:**
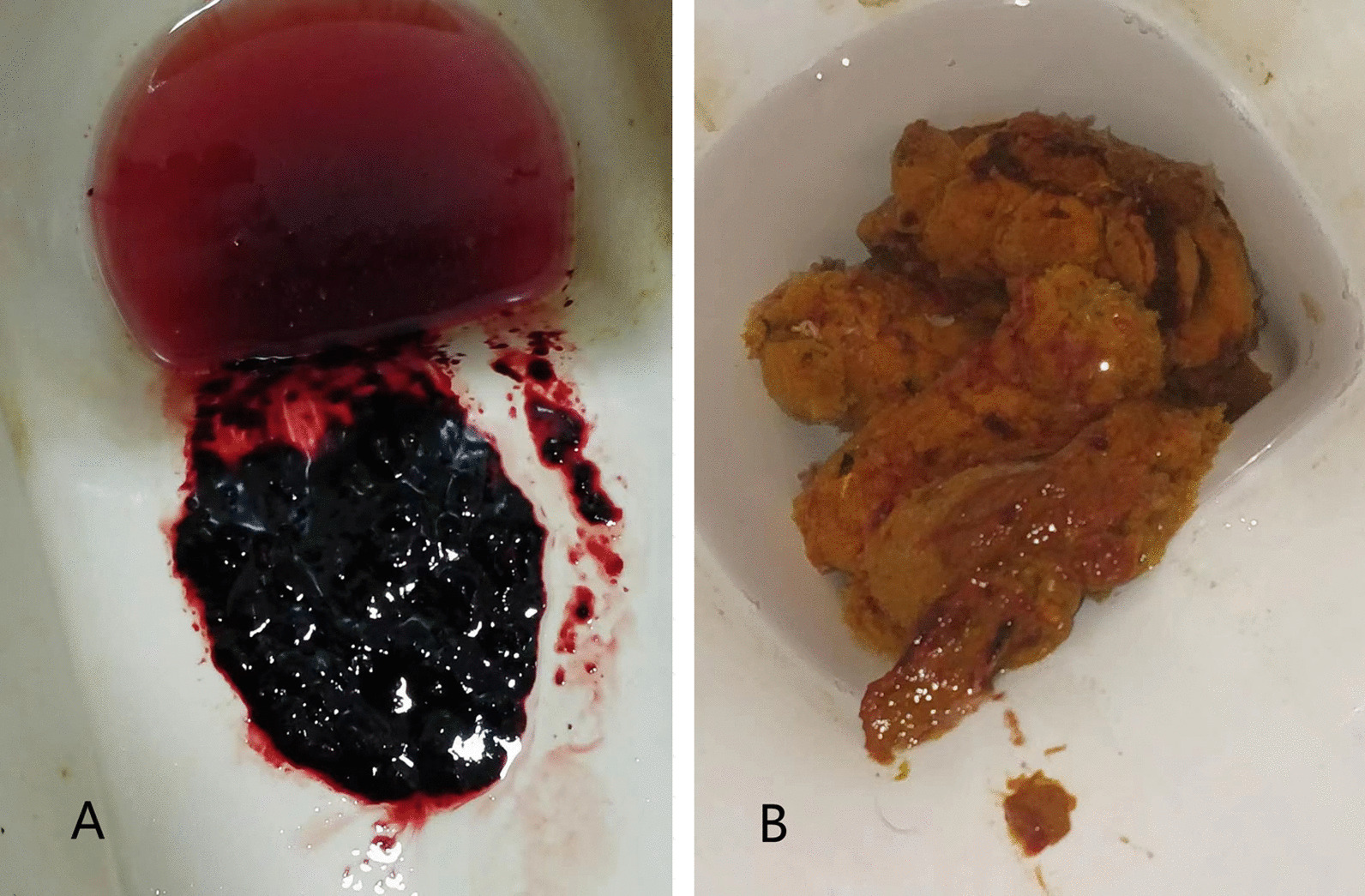
Grade 3 treatment-related adverse events. **A** Hematochezia after two cycles of the combined therapy. **B** Hematochezia disappeared after active management

Combined chemotherapy (vincristine 2 mg D1, epirubicin 100 mg D1, ifosfamide 2000 mg D1-5 and mesna 400 mg t.i.d D1-5, q3w) was started in July 2019. The patient also received anlotinib at an initial dosage of 12 mg, once daily, 2-week on/1-week off. After two active cycles, abdominal CT indicated a partial response (PR) (according to the RECIST version 1.1) as multiple masses were largely reduced (Fig. 1D–F). However, the patient suffered grade 3 treatment-related adverse events (TRAE) including hematochezia (Fig. 3A). Anlotinib was discontinued due to concerns relating to the safety profile. Meanwhile, patient received oral carbazochrome sodium sulfonate, loperamide hydrochloride, as well as Chinese medicine, Yunnan Baiyao. With these active managements, hematochezia stopped 3 days later and continuous fecal occult blood tests were negative (Fig. 3B).

Considering the regression and necrosis of the tumor, as well as the anti-VEGFR effect of anlotinib that may have led to hematochezia, the dose of anlotinib was adjusted to 8 mg, once daily, 2-week on/1-week off and re-started in the third cycle. After the dose adjustment, hematochezia disappeared and the combined treatment was well tolerated. A total of 6 cycles were administrated and a follow-up CT scan indicated continuous PR (according to the RECIST version 1.1) (Fig. 1G–I).

Aggressive surgery requiring colostomy and ureterostomy was recommended by the MDT which has a serious impact on quality of life (QOL). The young man refused surgical resection and received anlotinib as maintenance therapy. During maintenance therapy, the most common grade 3 TRAE (according to the CTCAE version 5) were proteinuria and hypertension, especially on the first 14-days of the continuous dose schedule. Notably, when anlotinib was discontinued for 7 days, the symptoms regressed. Based on this response, a 1-week on/1-week off regimen was adopted (8 mg, once daily). The dose reduction and active management of toxicities resulted in the maintenance therapy being well-tolerated. A follow-up abdominal CT in August 2020 indicated continuous PR (according to the RECIST version 1.1). Treatment with anlotinib was continued without grade 3 or 4 TRAE. However, the latest abdominal CT in June 2021 suggested progressive disease (PD) (according to the RECIST version 1.1). As the patient refused to restart chemotherapy, immunotherapy combined with anlotinib was recommended.

## Discussion and conclusions

IADSRCT is a rare neoplasm in young adult patients that most commonly occurs in males. The typical presentation is an intra-abdominal primary mass associated with visceral and parietal seeding of the peritoneum and the disease progresses rapidly [9]. The diagnosis of IADSRCT is based on the histologic appearance of samples and has been previously described by Gerald et al. The disease is characterized by small round cells with a scarce cytoplasm and the presence of occasional fibroblast-like cells. Positive immunostaining of cytokeratin, desmin and NSE can also contribute to the accurate diagnosis of IADSRCT [10].

Multidisciplinary treatment is the cornerstone of IADSRCT management. Standard chemotherapy consists of vincristine, doxorubicin, cyclophosphamide, ifosfamide, etoposide and dactinomycin. Complete surgical resection and radiation therapy can improve the survival of patients with IADSRCT [2]. Also, the treatment landscape for DSRCT has recently evolved with the implementation of tyrosine kinase inhibitors.

We reviewed the literature and summarized the cases of DSRCT with detailed medical information. The characteristic of these studies was summarized in Table 1. Ramspott et al. reported a case involved an elderly patient, who received laparoscopy and palliative chemotherapy. Unfortunately, only a few weeks later, the patient died due to cardiovascular failure [11]. Shi et al. observed an outstanding efficacy of apatinib monotherapy on reducing mass size and ascites in a young man with DSRCT [12]. A case report by Tian et al. confirmed the efficacy of apatinib in combination with chemotherapy as a second-line treatment for a patient with DSRCT with the best response being PR [13]. Also, Ikeue et al. found that treatment with pazopanib significantly reduced tumor burden after the failure of salvage chemotherapy in a 32-year-old Japanese man with DSRCT [14]. Thijs et al. [15] evaluated the mTOR‐inhibitor Temsirolimus in DSRCT. As a third-line therapy, the response to temsirolimus was very promising and lasted for over 40 weeks. Despite particular case reports, the efficacy and safety of targeted therapies for DSRCT have mostly been evaluated in retrospective studies. For example, Frezza et al. reviewed the data of patients with metastasis DSRCT who received pazopanib as subsequent treatment. The combined analysis indicated that pazopanib was associated with clinical benefits in these patients [16]. A retrospective analysis conducted by Italiano et al. also revealed that sunitinib provided promising efficacy in patients with heavily pretreated DSRCT [17].

**Table 1:** Summary of the cases of DSRCT

References	Gender	Age	Site of primary tumor	Surgery	Targeted therapy	Treatment line	Other therapy^a^	Treatment response
[11]	Male	69	Peritoneal cavity	Yes	No	NA^b^	Yes	NA^b^
[12]	Male	32	Mesentery	Yes	Apatinib	2nd	No	PR
[13]	Male	31	Abdominal cavity	Yes	Apatinib	2nd	Yes	PR
[14]	Male	32	Pleural surface	Yes	Pazopanib	2nd	No	PR
[15]	Male	21	Pelvis	No	Temsirolimus	3rd	No	SD
[18]	Male	38	Peritoneal cavity	Yes	Anlotinib	1st	No	SD
Our case	Male	27	Pelvic and abdominal cavity	No	Anlotinib	1st and mainteiance	Yes	PR

^a^Including chemotherapy and/or radiotherapy

^b^Not mentioned

By inhibiting tumor growth and angiogenesis, anlotinib has shown encouraging efficacy in metastatic STS. Existing evidence indicates that chemotherapy combined with anlotinib plus anlotinib maintenance therapy can induce objective responses in advanced/metastatic STS [7, 8]. However, limited evidence of the benefit of anlotinib in IADSRCT has been presented only by Chen et al. [18]. In contrast to the patient in this report, a 38-year-old man with IADSRCT received laparoscopic tumor resection with anterior abdominal wall nodule resection and 6 cycles of adjuvant chemotherapy (ifosfamide + liposomal doxorubicin). Unfortunately, the patient progressed with metastasis to the right inguinal and omental lymph nodes. Anlotinib was administered and showing promising efficacy as the lymph nodes were significantly reduced after four cycles. This novel strategy indicated that anlotinib could control progressive disease in patients with IADSRCT. It also provides a rationale to evaluate the treatment in young patients with IADSRCT with good performance status (PS) by adding anlotinib to the backbone chemotherapy.

The patient in our study had extensive metastasis at initial diagnosis and so the intensive combination regimen was recommended as a neoadjuvant treatment aiming to obtain complete resection after maximum tumor regression. When managed with the combined therapy, multiple masses in the patient were significantly reduced and a continuous PR was achieved. Also, the lesions continuously regressed during the maintenance treatment. Overall, the duration of response (DOR) lasted for nearly 2 years.

For combined regimens, safety and treatment tolerance are critical. In our study, the most common AEs were hematochezia, proteinuria and hypertension which were in line with previous studies [6–8]. Severe hematochezia has not been frequently reported following the administration of anlotinib. The patient in our study suffered grade 3 hematochezia after 2 cycles of the combined treatment which may have been caused by regression of the tumor and the anti-VEGFR effect of anlotinib. Anlotinib was discontinued for several days due to safety concerns. When the TRAE disappeared, anlotinib was re-started at a lower dose. The positive management of TRAE and the individual dose-adjustment of anlotinib were effective as the subsequent treatments were well-tolerated and a continuous PR was achieved. This provides experience and an instructive lesson for combined therapy in patients with IADSRCT with heavy tumor burdens. The preventive management of hemostasis and other predictable toxicities may play critical roles during the treatment.

Overall, our study confirmed the value of chemotherapy combined with anlotinib plus anlotinib maintenance in a patient with advanced IADSRCT. The management of AEs is critical to successful outcomes. There is a lack of prospective clinical trials for this rare disease and the findings reported in this study require further validation in large patient cohorts.

## Data Availability

Not applicable.

## Abbreviations

IADSRCT: Intra-abdominal desmoplastic small round cell tumor
STS: Soft-tissue sarcomas
DSRCT: Desmoplastic small round cell tumors
TKI: Tyrosine kinase inhibitor
ORR: Objective response rate
DCR: Disease control rate
CT: Computer tomography
MDT: Multidisciplinary team
PR: Partial response
TRAE: Treatment-related adverse events
QOL: Quality of life
PD: Progressive disease
PS: Performance status
